# The Interplay of Seniority and Superiority Toward Perceived Employability of Older Workers Through a Cultural Lens

**DOI:** 10.1093/geroni/igaa057.215

**Published:** 2020-12-16

**Authors:** Clio Y M Cheng, Vivian Lou

**Affiliations:** The University of Hong Kong, Hong Kong, China

## Abstract

Background: Hong Kong will become a super-aged society comprising more than 21% of its citizens aged 65 or above by 2024. With longer life expectancy and better health conditions of the elderly, the mentality of embracing “hidden gems” - older workers was under the spotlight. Extended working life provided a golden chance for employers and/or human resources (HR) personnel to manage this demographic change. Sau Po Centre on Ageing was commissioned to initiate a study in 2017 to 2018 on how employers and/or HR personnel perceived employability of older workers in Hong Kong, and to consolidate good practices of elder-friendly employment. Methods: Aiming to garner opinions from a wide variety of employers and/or HR personnel, 33 in-depth interviews were conducted with participants from various industries and company sizes, including both large corporations and small and medium enterprises (SMEs). Among the participants, nearly half (n=15) possessed managerial position, other including employers (n=1), directors (n=6), officers (n=8) and others (n=3). Among these companies, 19 did not have any mandatory retirement age policies, while seven had their retirement age set as 60 years old, and another seven set their retirement age at aged 65. Results: Participants tended to link seniority, in terms of both chronological age and loyalty, with superiority, affecting their perceived employability of older workers. This study has multi-level implications on a multi-generational age-inclusive workforce management strategy on recruitment, retainment and retraining. Suggestions on good practices of an age-inclusive and age-diverse workforce were also made at individual, corporation and societal level.

